# Multimodal analysis of *Plasmodium knowlesi*‐infected erythrocytes reveals large invaginations, swelling of the host cell, and rheological defects

**DOI:** 10.1111/cmi.13005

**Published:** 2019-02-11

**Authors:** Boyin Liu, Adam J. Blanch, Arman Namvar, Olivia Carmo, Snigdha Tiash, Dean Andrew, Eric Hanssen, Vijay Rajagopal, Matthew W.A. Dixon, Leann Tilley

**Affiliations:** ^1^ Department of Biochemistry and Molecular Biology Bio21 Molecular Science and Biotechnology Institute Melbourne Victoria Australia; ^2^ Department of Biomedical Engineering The University of Melbourne Melbourne Victoria Australia; ^3^ Advanced Microscopy Facility Bio21 Molecular Science and Biotechnology Institute, The University of Melbourne Melbourne Victoria Australia

## Abstract

The simian parasite *Plasmodium knowlesi* causes severe and fatal malaria infections in humans, but the process of host cell remodelling that underpins the pathology of this zoonotic parasite is only poorly understood. We have used serial block‐face scanning electron microscopy to explore the topography of P. knowlesi‐infected red blood cells (RBCs) at different stages of asexual development. The parasite elaborates large flattened cisternae (Sinton Mulligan's clefts) and tubular vesicles in the host cell cytoplasm, as well as parasitophorous vacuole membrane bulges and blebs, and caveolar structures at the RBC membrane. Large invaginations of host RBC cytoplasm are formed early in development, both from classical cytostomal structures and from larger stabilised pores. Although degradation of haemoglobin is observed in multiple disconnected digestive vacuoles, the persistence of large invaginations during development suggests inefficient consumption of the host cell cytoplasm. The parasite eventually occupies ~40% of the host RBC volume, inducing a 20% increase in volume of the host RBC and an 11% decrease in the surface area to volume ratio, which collectively decreases the ability of the P. knowlesi‐infected RBCs to enter small capillaries of a human erythrocyte microchannel analyser. Ektacytometry reveals a markedly decreased deformability, whereas correlative light microscopy/scanning electron microscopy and python‐based skeleton analysis (Skan) reveal modifications to the surface of infected RBCs that underpin these physical changes. We show that P. knowlesi‐infected RBCs are refractory to treatment with sorbitol lysis but are hypersensitive to hypotonic lysis. The observed physical changes in the host RBCs may underpin the pathology observed in patients infected with P. knowlesi.

## 1 INTRODUCTION

*Plasmodium knowlesi* is a parasite of long‐tailed macaques (Macaca fascicularis) that causes zoonotic infections in humans (Chin, Contacos, Collins, Jeter, & Alpert, 1968). It is the most common cause of malaria in East Malaysia, and the mosquito vectors for P. knowlesi are present throughout Southeast Asia, where an estimated 500 million people are at risk of infection (Barber, Rajahram, Grigg, William, & Anstey, 2017; Shearer et al., 2016; William et al., 2014). The blood stages of P. knowlesi are responsible for disease pathology, and high parasitemia infections can cause severe malaria in adult humans at a rate similar to that of Plasmodium falciparum (Barber et al., 2013; Cox‐Singh & Singh, 2008). Anaemia and thrombocytopenia (low platelet count) are frequently observed, along with impaired renal and liver function (Barber et al., 2011; Daneshvar et al., 2009; Singh & Daneshvar, 2013).

P. knowlesi lacks the virulence protein family, P. falciparum erythrocyte membrane protein‐1 (*Pf*EMP1), that mediates cytoadherence of P. falciparum‐infected red blood cells (RBCs), but expresses another variant surface antigen named schizont‐infected cell agglutination antigens (al‐Khedery, Barnwell, & Galinski, 1999; Howard, Barnwell, & Kao, 1983). Although evidence for adhesion of P. knowlesi‐infected RBCs is limited (Fatih et al., 2012), a recent study using ektacytometry and micropipette aspiration revealed increased rigidity of infected and uninfected RBCs in samples collected from adult patients with severe knowlesi malaria (Barber et al., 2018). Infection of rhesus macaques (Macaca mulatta), an unnatural host for P. knowlesi, leads to impaired microcirculatory flow, causing severe and fatal disease (Knisely, Stratman‐Thomas, Eliot, & Bloch, 1964). Moreover, an autopsy of a fatal human knowlesi malaria case revealed brain capillary congestion (Menezes et al., 2012).

The potential for trapping or sequestration of mature stage P. knowlesi‐infected RBCs in sites away from the peripheral circulation is indicated by the reported decrease in the percentage parasitemia in peripheral blood in P. knowlesi infections in rhesus macaques as the parasites mature from ring to schizont stage (Miller, Fremount, & Luse, 1971). The same study showed accumulation of schizont‐infected RBCs in the hepatic sinusoids and in the venules of the small intestine. At higher parasitemia levels, parasitised RBCs were found in other organs, including cerebral capillaries and venules (Miller et al., 1971). These early studies indicate that occlusion of small capillaries by trapped P. knowlesi‐infected RBCs could underpin the pathology of P. knowlesi in human infections; however, the mechanism of trapping remains unclear.

P. knowlesi has been successfully adapted for sustained culture in human RBCs (Gruring et al., 2014; Moon et al., 2013), providing an important model for this emerging zoonotic pathogen. This provides an opportunity to undertake a detailed ultrastructural and biomechanical analysis of the development of P. knowlesi in human RBCs. Using a multimodal approach, we observed a marked accumulation of haemoglobin‐containing invaginations, novel deposits at the RBCs membrane, substantial host cell swelling, and altered rheological properties, which may collectively contribute to the pathology of P. knowlesi *‐*infected human RBCs.

## 2 RESULTS

**Figure 1:**
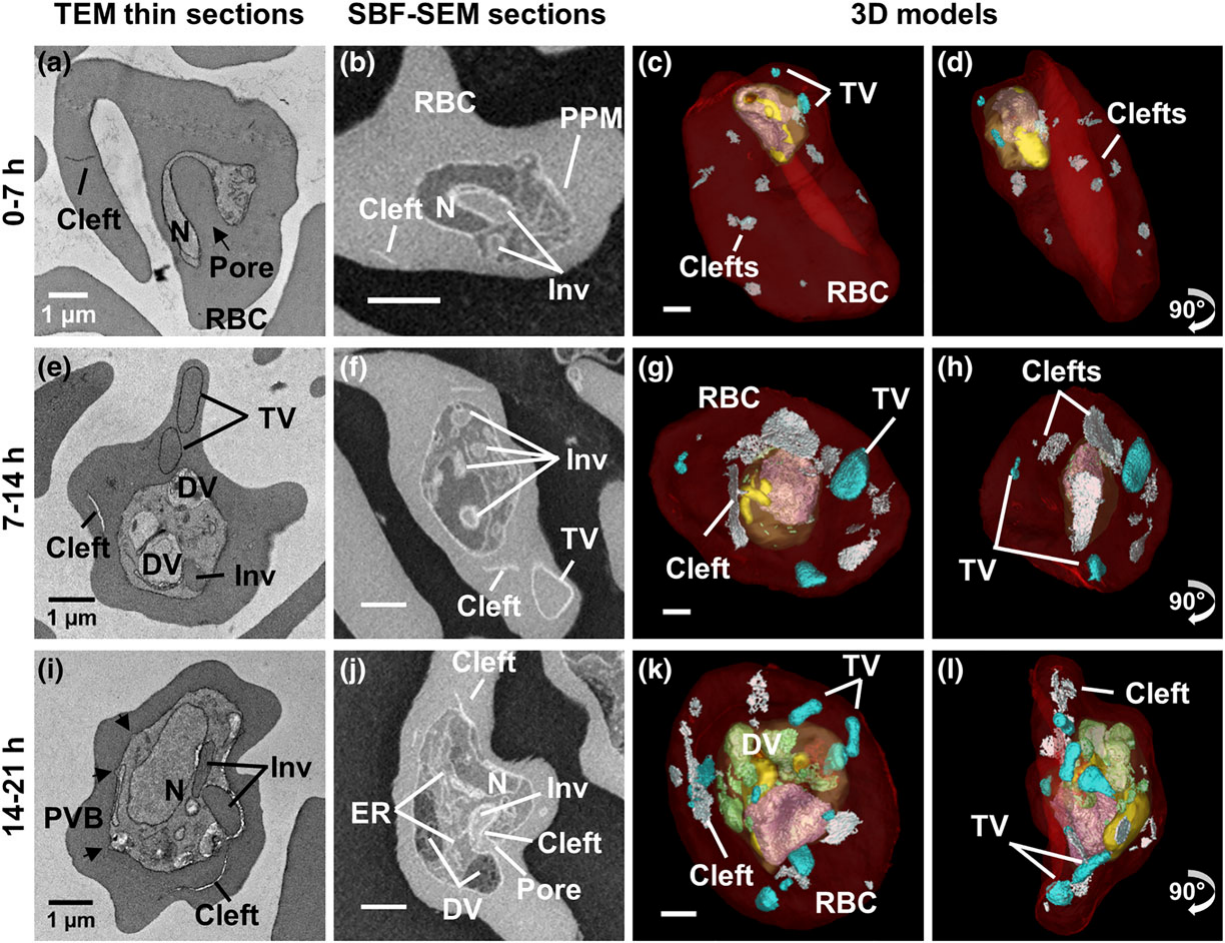
Ultrastructure of P *lasmodium* knowlesi at ring and trophozoite stages. First column: transmission electron microscopy (TEM) of plastic‐embedded thin sections. Right hand columns: serial block‐face scanning electron microscopy (SBF‐SEM) images and rendered 3D models of the same cells. (a–d) 0 to 7‐hr postinvasion. (a) The arrow indicates the invagination pore. (b) The invagination (Inv) deforms the nucleus (N). (c–d) The early ring parasite occupies a small portion of the red blood cell (RBC). Small tubular vesicle (TV) and clefts are present in the RBC cytoplasm. (e–h) 7 to 14‐hr postinvasion. (e) A digestive vacuole (DV) is evident indicating initiation of haemoglobin digestion. (f) A branched invagination is observed. (g–h) Hemozoin crystals (green) are evident. Larger TV are observed. (i–l) 14 to 21‐hr postinvasion. Parasitophorous vacuole bulges (PVB) have formed (black arrows) and a pore in (j) indicates active haemoglobin uptake. (k–l) Larger DVs (green) accumulate next to the invaginations. Colours: PVM, pale yellow; nuclei, pink; invagination, yellow; DV, green; RBC, translucent red; clefts, white; TV, cyan. Panels on the far right represent 90^o^ rotations of the preceding panels (see Videos S1–S6 for translations and rotations of these cells)

**Figure 2:**
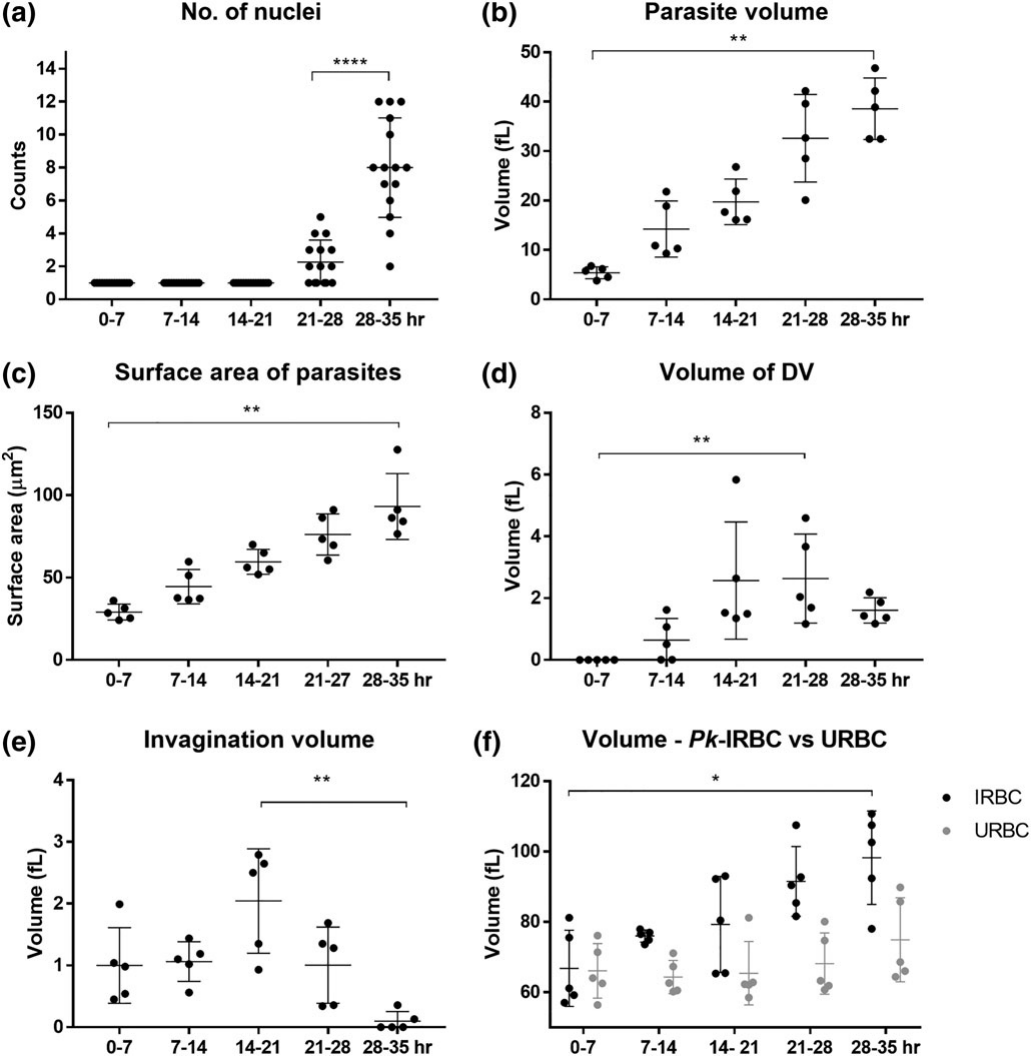
Quantification of P *lasmodium* knowlesi structural features. (a) Nuclear division is initiated at late trophozoite stage, forming up to 12 nuclei. (b,c) Parasite volume and surface area increase steadily during development. (d) Digestive vacuole (DV) volume peaks at trophozoite stage. The DVs fuse into one remnant body at late schizont stage. (e) Invagination size reaches a maximum at trophozoite stage. No haemoglobin uptake occurs in late schizont stage. (f) The infected red blood cell (RBC) volume increases with parasite age. Data represent mean values and standard errors. Unpaired *t* test; *p < 0.1; **p < 0.01; ****p < 0.0001

**Figure 3:**
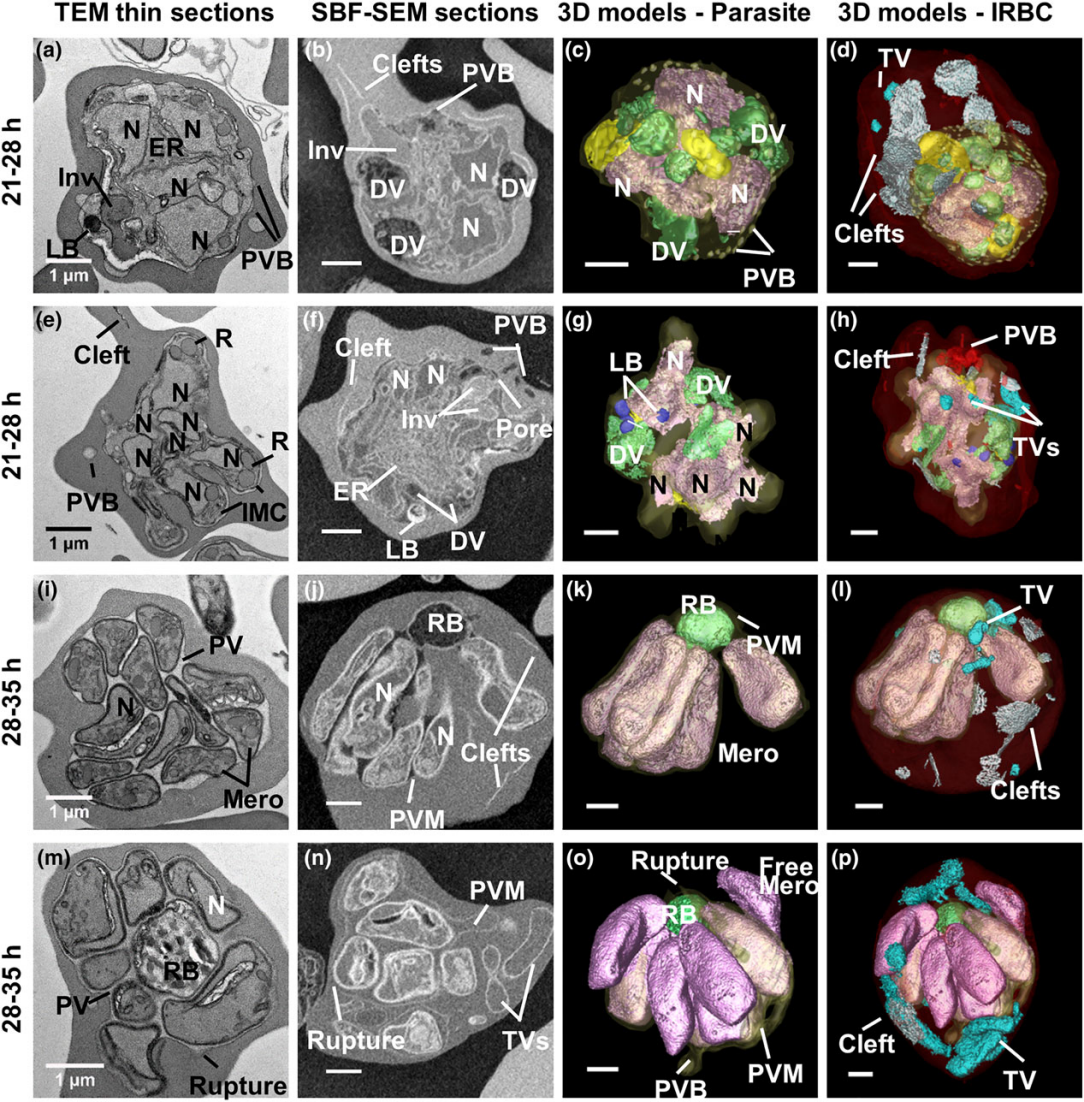
Ultrastructure of P *lasmodium* knowlesi at the early schizont and segmenter stages. First column: transmission electron microscopy (TEM) images. Second to fourth columns: serial block‐face scanning electron microscopy SBF‐SEM images and rendered 3D models of the same cells. (a–h) Schizonts with 3–4 (a–d) or ~8 (e–h) nuclei. (a–h) Large digestive vacuoles (DVs), depleted invaginations and electron‐dense lipid bodies (LB) are evident. (b,c) Parasitophorous vacuole bulges (PVB, rendered in white) are evident. (e) In a more developed schizont, the apical end of the nascent daughter merozoites deform the mother cell and the developing inner membrane complex (IMC) and rhoptries (R) are evident. (f) The schizont is very rich in ER. The invagination pore is still evident. (g) Five protrusions of the PV are evident as the developing merozoites extend. (f,h) A cluster of PV blebs (red) is evident in the host red blood cell (RBC) cytoplasm. (i–p) Segmentation of the individual merozoites can be seen. (i–l) The plasma membrane forms around individual nascent merozoites, but they remain attached at the basal end to the remnant body (RB). The PV lumen remains electron lucent. (l) The whole cell model reveals the persistence of exomembrane structures. (m–p) A remnant body full of hemozoin crystals is surrounded by fully formed merozoites. Rupture of the parasitophorous vacuole membrane (PVM) permits leakage of RBC haemoglobin into the PV. (o) The rendered 3D model shows PVM rupture at multiple locations and one detached merozoite. (p) Colours: PVM, pale yellow; nuclei, pink; invaginations, yellow; DV/RB, green; LB, deep blue; RBC, translucent red; clefts, white; tubular vesicle (TV), cyan; PV bulges, fawn; PV blebs, red (see Videos S7–S12 for translations and rotations of these cells)

We found that P. knowlesi A1 strain developing in human RBCs in culture exhibits an intraerythrocytic cycle of ∼32‐hr duration, in reasonable agreement with a previous report (Moon et al., 2013). Using a magnet purification/invasion/magnet depletion regime (see methods), we synchronised P. knowlesi cultures to windows of 7 hr and collected samples at different stages of development. We analysed samples by conventional thin section transmission electron microscopy (TEM) and serial block‐face scanning electron microscopy (SBF‐SEM; Denk & Horstmann, 2004; Sakaguchi, Miyazaki, Fujioka, Kaneko, & Murata, 2016; Figures 1, 2, 3). We generated SBF‐SEM 3D volumes at ~50‐nm resolution. The cellular features are revealed in individual SBF‐SEM “sections,” and rendering these features provides a detailed topographic map of cellular organisation. The following features were identified: RBC membrane (RBC; red), parasitophorous vacuole (PV) membrane (PVM, transparent yellow), invaginations (yellow), PV bulges (fawn), nucleus (N, pink), digestive vacuoles (DV; green), Sinton Mulligan's clefts (white), PV blebs (red), lipid bodies (deep blue), and tubular vesicles (TV; cyan). See Videos S1–S12 for translations through the individual sections and rotations of the rendered models (two example cells for each stage are shown).

### 2.1 3D ultrastructure of the ring and trophozoite stages of P. knowlesi

In the earliest stages of development (0 to 7‐hr postinvasion), the parasite adopts a bowl shape, with invagination of regions of host cell cytoplasm already apparent in some cells (Figure 1a–d). At this stage, Sinton Mulligan's clefts and TV are already evident in the RBC cytoplasm (rendered respectively in white and cyan, Figure 1c,d). The parasite has an average volume of 5 ± 1 fl (Figure 2b) and a relatively high surface area to volume ratio (Figure S1f). The organisation of the different structures is best appreciated in Videos S1 and S2. We note that the infected RBC volume is equivalent to that of uninfected RBCs (~68 fl; Figure 2f).

Upon maturation to the early trophozoite stage (7‐ to 14‐hr postinvasion), a more flattened parasite shape is observed, and the invaginations of the host RBC into the parasite become more elaborate (Figure 1e–h; Videos S3 and S4). Commencement of haemoglobin digestion is evidenced by the appearance of small dispersed hemozoin‐containing structures (Figure 1g,h; rendered in green); however, no mature (fused) DV compartments are evident at this stage. The exomembrane features increase in prominence (Figures 1g,h and S1b,c). The parasite has an average volume of 14 ± 6 fl, and the infected RBC exhibits a slightly increased volume (~75 fl; Figure 2f).

Mature trophozoites (14‐ to 21‐hr postinvasion) still have a single nucleus but exhibit increased thickness and volume (Figures 1i–l, 2, and S1e and Videos S5 and S6). The endoplasmic reticulum (ER) is prominent (labelled on the figure), consistent with augmented production of proteins and membrane. The invaginations of the RBC cytoplasm persist, reaching peak volume (Figure 2e), while mature DV (containing hemozoin crystals; green) become prominent. The parasite has an average volume of 20 ± 6 fl, and the infected RBC exhibits a further increase in volume (~80 fl; Figure 2f).

### 2.2 3D ultrastructure of the schizont stage of P. knowlesi

In the early schizont stage (21 to 28‐hr postinvasion), nuclear division has been initiated (average number of nuclei = 2; range 1–6; Figures 3a–h and 2a; Videos S7 and S8). The DV (green in Figure 3c,d,g,h) increase in prominence and reach peak total volume (Figure 2d). A prominent feature of this stage is bulges of the PVM (i.e., areas of separation of the parasite plasma membrane (PPM) and PVM; Figure 3c, rendered in fawn). Densely staining features are evident in the parasite cytoplasm (Figure 3a,g,h; rendered in deep blue). These are likely neutral lipid bodies involved in lipid storage (Jackson et al., 2004). The PVM becomes distended around the nascent daughter merozoites as the nucleus migrates to the basal end (Figure 3e,g). The parasite reaches an average volume of 32 ± 6 fl (Figure 2b), giving a lower surface area to volume ratio (Figure S1f). The total volume of the infected RBC has substantively increased (~86 fl; Figure 2f), and the parasite occupies ~30% of the host cell volume.

In mature segmented schizonts (28 to 35‐hr postinvasion), the average number of nuclei increases to 8 (range 2–12; Figure 2a; Videos S9–S12). Odd numbers of nuclei are evident in some cells indicating asynchronous nuclear division. Individual daughter merozoites are evident within a tightly encasing PVM (Figure 3i–p). The individual parasites remain connected to the remnant body (RB), which contains the remnant DV material. No RBC cytoplasmic invaginations are evident, indicating that haemoglobin digestion is completed. In some schizonts, the PV lumen has a lower density than that of the RBC cytoplasm (Figure 3i,j,m), indicating that the PVM remains intact. In others, the region surrounding the daughter merozoites has a density equivalent to that of the RBC cytoplasm (Figure 3n), indicating that the barrier properties of the PVM have been compromised. Indeed, rendering of this cell shows that the PVM has been breached (Figure 3o). The average parasite volume is 38 ± 4 fl (Figure 2b). The infected RBC has a substantively increased total volume (~100 fl; Figure 2f), with the parasite occupying ~40% of the host cell volume.

### 2.3 Merozoites undergo morphology changes upon release from the schizont

**Figure 4:**
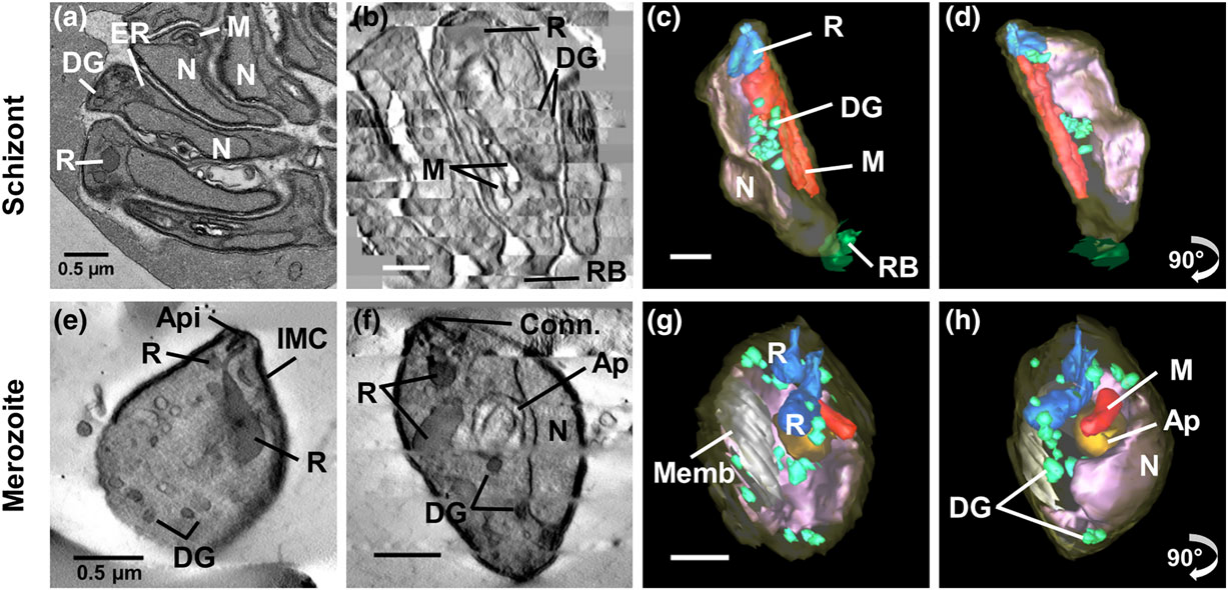
High‐resolution tomograms and 3D models of P *lasmodium* knowlesi segmenter merozoites and free merozoites. Transmission electron microscopy (TEM) image, stack of virtual sections, and 3D models of segmenter merozoites (a–d) and free merozoites (e–h). (b,e,f) Virtual sections through schizont segmenter merozoites (b) and free merozoites (e,f) showing rhoptries (R), dense granules (DG), mitochondria (M), apicoplast (Ap), stacked membranes (memb), remnant body (RB) and nuclei (N). (c,d,g,h) Models of a segmenter merozoite (c,d) and free merozoites (g,h) showing merozoite PM, translucent yellow; rhoptries, blue; dense granules, green; mitochondria, red; apicoplast, orange; and nuclei, pink. Panels on the far right represent 90^o^ rotations of the preceding panels (see Videos S13 and S14 for translations and rotations of these cells)

The schizont merozoites are quite elongated (length 3.6 ± 0.3 μm, width 1.2 ± 0.3 μm) and exhibit an average volume of 3.8 ± 0.2 fl (*n* = 18 merozoites from two schizonts with ruptured PVM), whereas the free merozoites are shorter (length 3.0 ± 0.2 μm, width 1.5 ± 0.1 μm) with an average volume of 3.4 ± 0.5 fl (*n* = 11). Individual ultrastructural features of merozoites are difficult to discern at the level of resolution afforded by SBF‐SEM. We therefore undertook electron tomography of multiple sections of schizonts and free merozoites (see Figure 4b,e,f for virtual sections from the joined stacks). Merozoites are characterised by a large elongated nucleus and prominent apical organelles. A pair of flask‐shaped rhoptries with the typical neck and bulb features are observed, along with additional electron‐dense features (blue‐green) that are likely dense granules. The apicoplast and mitochondrion are closely associated. Stacked membranes (Memb) are evident in the merozoite cytoplasm. The organisation of the different structures is best appreciated in Videos S13 and S14.

### 2.4 Uptake of the host cell cytoplasm involves cytostomal invaginations and phagotrophs

**Figure 5:**
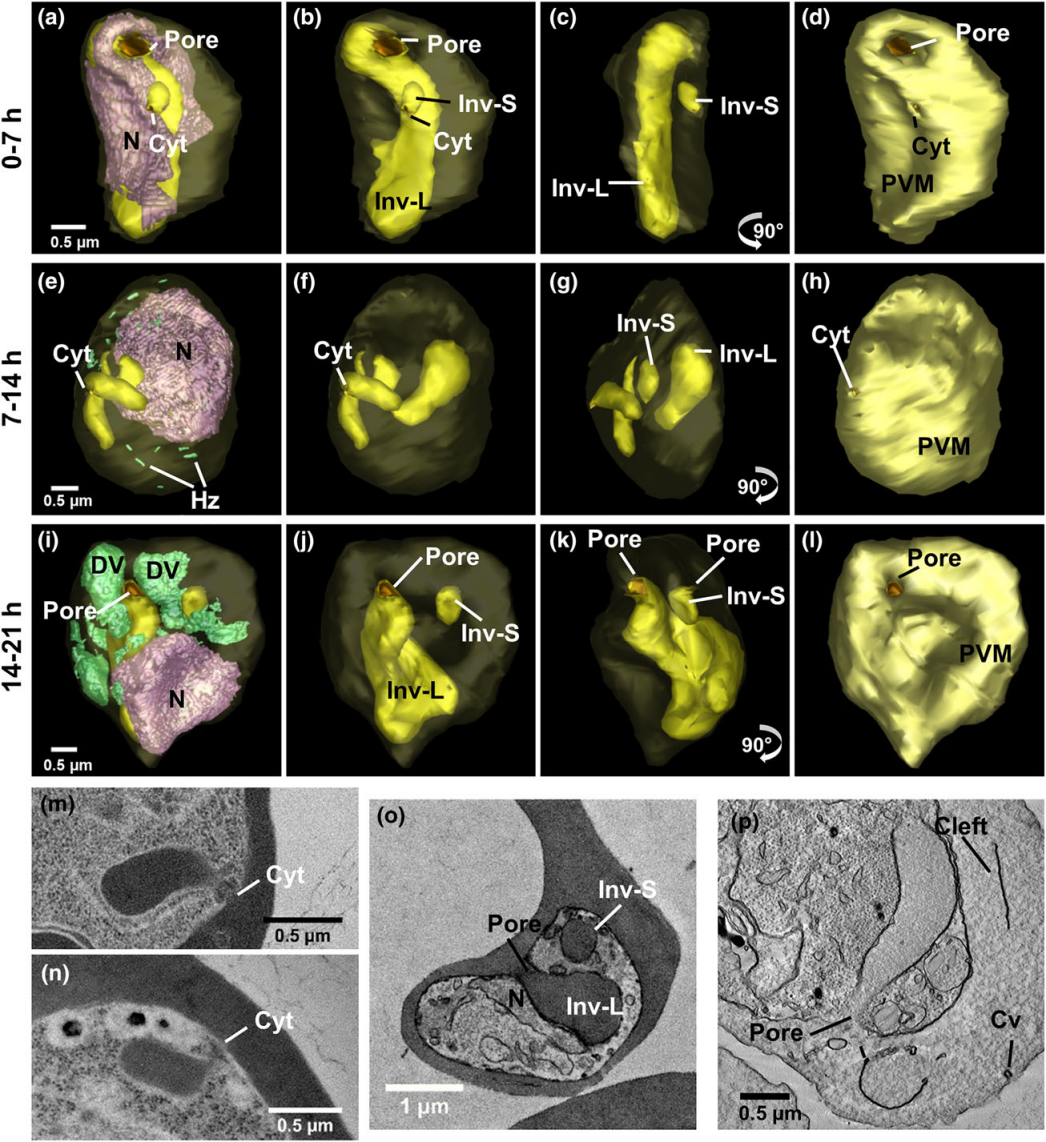
Ultrastructural analysis of invagination features. Rendered serial block‐face scanning electron microscopy (SBF‐SEM) models of (a–d) early ring stage, (e–h) late ring stage, (i–l) and trophozoite stage. (m–n) Transmission electron microscopy (TEM) images of cytostomes in late ring/trophozoite stage. The cytostome is a collar‐like structure with a diameter of 80–100 nm (m,n). (o,p) Large pores (~400 nm) connect invaginations to the host red blood cell (RBC) cytoplasm. (p) Haemoglobin is depleted from the invagination upon equinatoxin II (EqtII) permeabilisation

We surveyed structures involved in the uptake of host cell cytoplasm at different stages of development. As described above, soon after invasion, invaginations of the RBC cytoplasm into the parasite cytoplasm are observed (Figure 5, rendered in deep yellow). Closer examination of the rendered model reveals that the nucleus of the parasite wraps around the invaginations (Video S1). The invaginations remain connected to the bulk RBC cytoplasm by one of two different openings. Some invaginations are connected via small openings that likely represent classical cytostomes (Figure 5a–h, Cyt). Examples of active classical cytostomes are also observed in thin sections (Figure 5m,n). The cytostome comprises an electron‐dense ring with an internal diameter of ~90 nm and outer diameter of ~120 nm. Other (often larger volume) invaginations are connected to the bulk RBC cytoplasm via an opening with a diameter of ~400 nm. This is inconsistent with a cytostomal structure and is likely equivalent to the ~400 nm openings that are observed in thin sections (Figure 5o,p).

Both the smaller and larger invaginations remain in physical continuum with the host RBC cytoplasm, as indicated by equivalent densities (level of heavy metal binding) of the connected compartments. Moreover, when the infected RBCs are treated with equinatoxin II (EqtII), a pore‐forming toxin that selectively breaches the RBC membrane (Jackson et al., 2007), haemoglobin is lost from the invagination and the RBC cytoplasm, demonstrating that these compartments are connected (Figure 5p). As mature DVs (containing hemozoin crystals) are formed (Figure 5i, rendered in green), some DVs remain closely opposed to the two haemoglobin‐containing invaginations, suggesting that they are derived by pinching off regions of the invaginations. The presence of two different structures suggests two different modes of uptake of haemoglobin, which we refer to as cytostomal endocytosis and phagotrophic endocytosis.

### 2.5 The exomembrane system comprises a range of structural features

**Figure 6:**
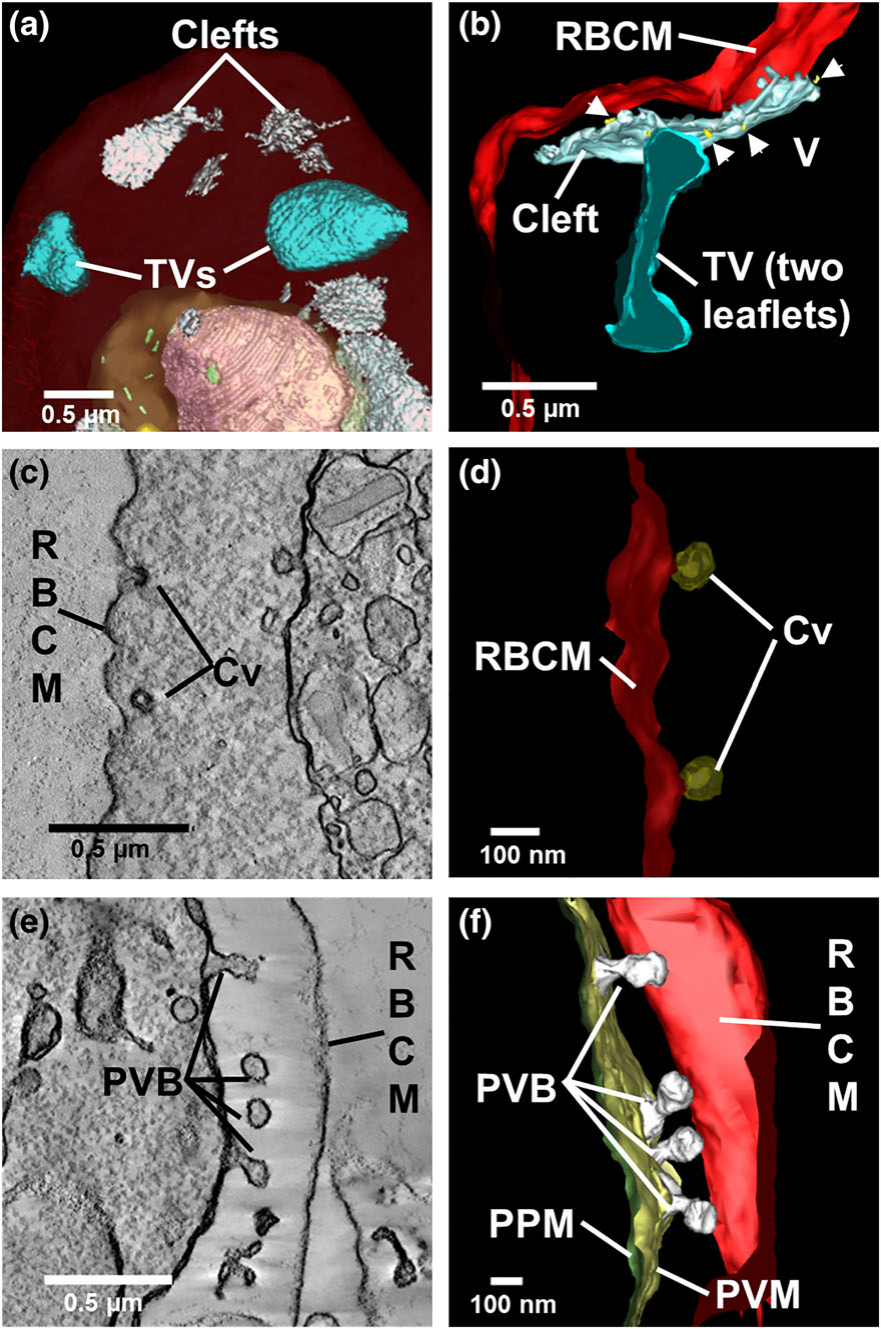
Detailed views of rendered clefts and tubular vesicle (TV). (a,b) Rendered serial block‐face scanning electron microscopy (SBF‐SEM; a) and electron tomography (b) models showing Sinton Mulligan's clefts (white) and TV (cyan). Small vesicles (gold) appear to bud from the cleft. (c,d) Virtual section through (c) and rendered model of (d) caveolae (Cv, bronze) at the host RBC membrane. (e,f) Virtual section through (e) and model of (f) membrane blebs (rendered in white) forming at the PVM

We made a detailed examination of the membrane features that P. knowlesi elaborates in the RBC cytoplasm, using SBF‐SEM and electron tomography (Figure 6). 10 to 15 narrow, slit‐like Sinton Mulligan's clefts are present from the earliest stages following invasion (clefts; ~1‐μm diameter; Figure 6a,b rendered in white; Figure S1c). The clefts are morphologically similar to, but larger than, the ~500‐nm Maurer's clefts of P. falciparum (Hanssen et al., 2010). Following depletion of host cell haemoglobin by selective permeabilisation with EqtII, it is clear that some clefts are decorated with small vesicular structures, suggesting budding or fusion (Figure 6b). Additional TV (~0.5‐μm diameter; Figure 6a,b, rendered in cyan) wrap around regions of RBC cytoplasm and are likely related to the tubulo‐vesicular network (TVN) complexes of P. falciparum (Haldar, Samuel, Mohandas, Harrison, & Hiller, 2001). The number of TV increases with age postinvasion, reaching an average of 8 (Figure S1d). We did not observe extensive networks of TV as has been reported for P. falciparum (Behari & Haldar, 1994) and for P. knowlesi developing in rhesus macaque RBCs (Kumi Asare et al., 2018).

Small indentations (80‐nm diameter) of the RBC surface, known as caveolae (Cv), are observed (Figure 6c). These are coated with a protein coat that stains with the heavy metals. Electron tomography reveals that these structures are roughly spherical in shape (Figure 6d).

In early schizonts, structures with an electron lucent lumen arise as bulges at the PV membrane (Figure 6e,f, rendered in white). The timing of the generation of these bulges (early schizont) is not consistent with their being involved in the formation of clefts or TV. Instead, they may be involved in expanding the parasite surface area, ahead of segmentation.

### 2.6 The host RBC membrane skeleton is stretched and decorated with deposits

**Figure 7:**
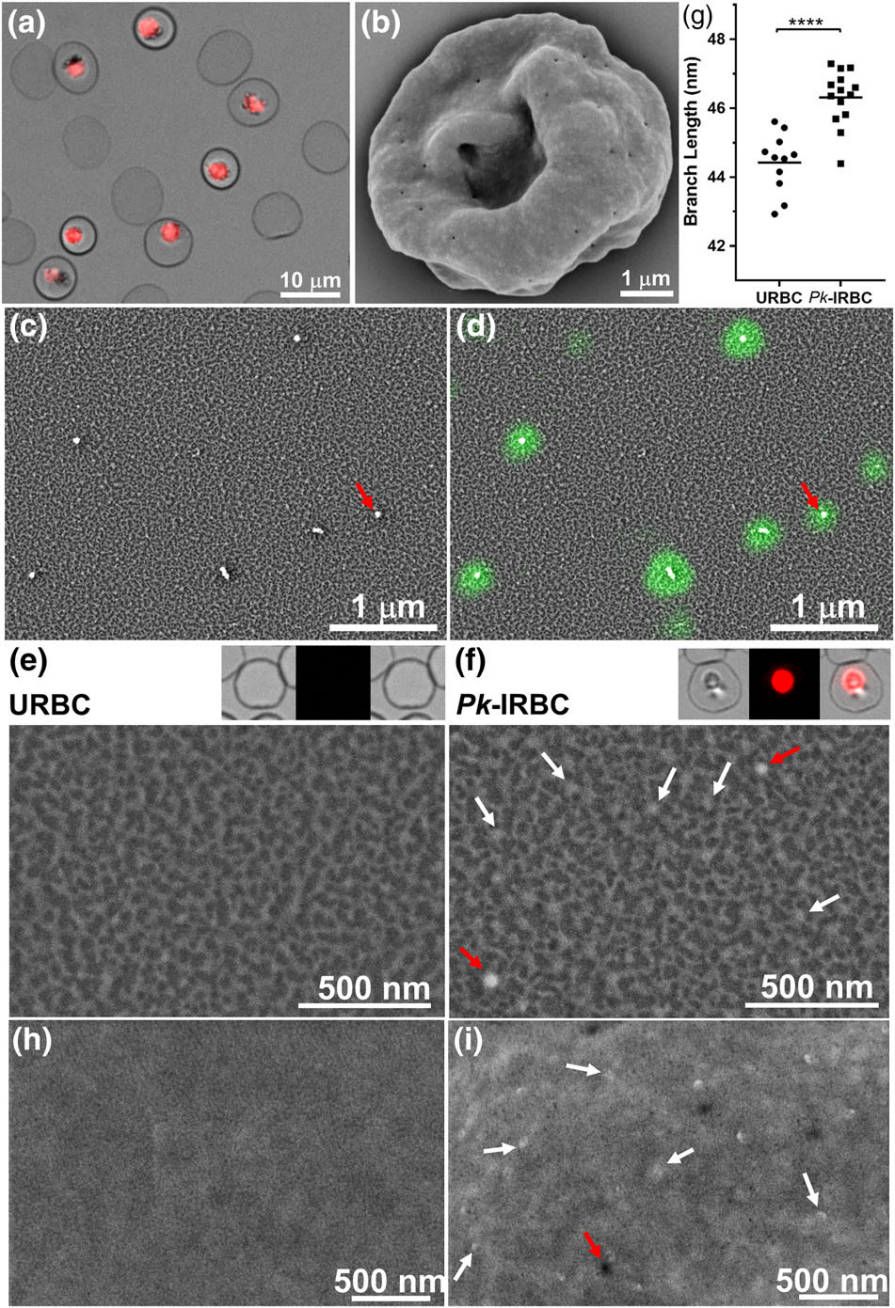
Structural modifications at the external and internal surfaces of *P. knowlesi*‐infected red blood cells (RBCs). (a) Late trophozoite stage P. knowlesi‐infected RBCs were immobilised onto glass slides and labelled with a nucleic acid‐binding dye. (b) Scanning electron microscopy (SEM) of a P. knowlesi trophozoite‐infected RBC. Shearing under hypotonic conditions leaves remnant membrane discs of (c,d,f) infected and (e) uninfected RBCs that were fixed, dehydrated, gold‐coated, and imaged using SEM. Caveolae were identified by labelling with *Pv*PHIST‐81 antibody followed by AlexaFluor647 staining, as overlaid in (d), appearing as bright electron‐scattering puncta. (e,f, insets) Light microscopy images before shearing of the RBCs whose membrane are depicted below. (h,i) High‐resolution SEM images of the external surface of (h) uninfected and (i) infected RBCs. (g) Average skeleton network branch distances were estimated using Skan. Representative 2.8 × 1 μm sections of external (whole cell) membranes imaged with backscatter SEM are shown for (h) uninfected and (i) infected RBCs

We established a correlative imaging method to probe the cytoplasmic surface of the host RBC. Late trophozoite stage P. knowlesi‐infected RBCs (~30% parasitemia) were immobilised onto marked glass slides that had been functionalised with (3‐aminopropyl)triethoxysilane and cross linked to the RBC binding lectin, phytohemagglutinin (Shi et al., 2013). The cells were labelled with the nucleic acid‐binding dye, Syto61, to identify infected RBCs (Figure 7a), before application of a stream of hypotonic buffer, leaving remnant RBC membranes bound to the glass slide.

The sheared membranes were fixed, dehydrated, and gold coated, and secondary and back‐scattered electrons were detected using high‐resolution SEM. The spectrin‐actin network appears as bright (raised) skeletal elements over dark patches of background (Figure 7c,d,e,f). Measurement of skeleton parameters from SEM images was performed using the *Skan* (skeleton analysis) python library—a fully automated method for selection and measurement of the skeleton network branch distances (Nunez‐Iglesias, Blanch, Looker, Dixon, & Tilley, 2018). Analysis of data from three separate experiments revealed an average branch distance of 44 ± 2 nm for uninfected RBC membranes, consistent with previous atomic force microscopy and cryoelectron microscopy‐based analyses (Nans, Mohandas, & Stokes, 2011; Shi et al., 2013) and our own previous analysis using SEM (Nunez‐Iglesias et al., 2018). We observed a moderate (4.5 ± 0.2%) increase in the length of the skeleton network branch length in infected cells (Figure 7g), indicating subtle reorganisation of the membrane skeleton.

P. knowlesi encodes a homologue (*Pk*PHIST‐105) of the caveolar‐vesicular complex protein, *Plasmodium vivax* PHIST/CVC‐81, for which an antiserum is available (Akinyi et al., 2012). The anti‐*Pv*PHIST‐81 antiserum recognises structures in sheared membranes that are separated by an average distance of 1.5 ± 0.6 μm (number of sheared membranes analysed = 6; Figures 7d and S2). Correlative imaging revealed that these fluorescently labelled structures correspond to raised punctate structures (red arrows, Figure 7c,d,f). In addition, smaller, more numerous punctate structures decorate the membrane skeleton of P. knowlesi‐infected RBCs (white arrows, Figure 7f), suggesting the deposition of parasite material onto the RBC membrane skeleton. We compared the images of the inner surface of the RBC membrane with SEM images of the infected RBC surface. The external surface exhibits two features. The Cv are observed separated by an average distance of 1.2 ± 0.6 μm (number of cells analysed = 6) and appear to have an invaginated aspect (Figure 7i, red arrows). We also observed more brightly scattering “pimples” (Figure 7i, white arrows), separated by an average distance of 0.3 ± 0.1 μm (*n* = 3 cells), which is similar to the punctate structures observed at the RBC membrane skeleton (average distance, 0.3 ± 0.1 μm; *n* = 4 cells). Taken together, the data are consistent with substantive modification of the human RBC membrane upon infection with P. knowlesi.

### 2.7 P. knowlesi‐infected RBCs are swollen and rigidified

**Figure 8:**
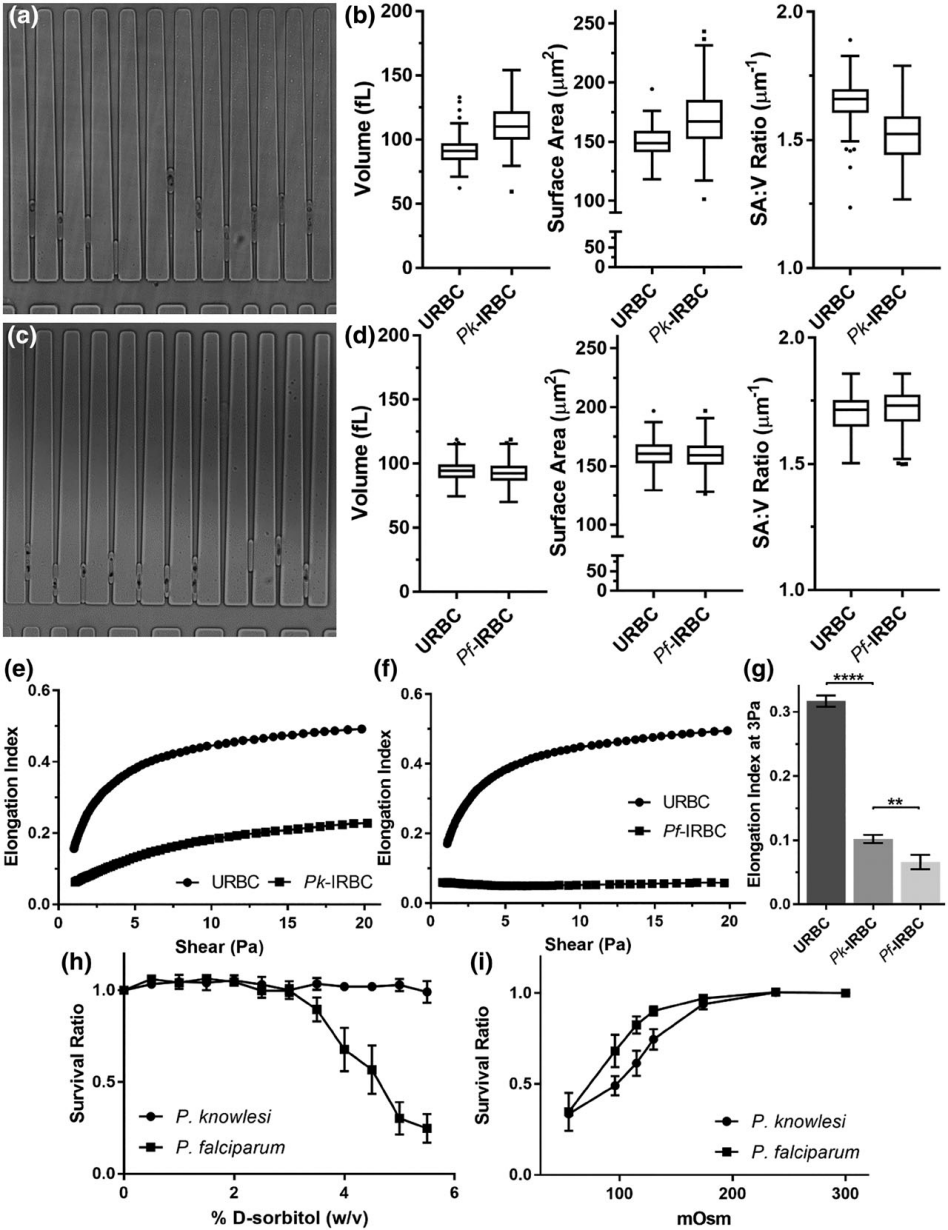
Rigidity, volume, and permeability properties of P *lasmodium* knowlesi and P *lasmodium* falciparum *‐*infected red blood cells (RBCs). (a,c) Images of the wedge‐shaped channels in the human erythrocyte microchannel analyser (HEMA) microdevice with trapped uninfected RBCs (URBCs) and P. knowlesi‐infected RBCs (*Pk*‐IRBCs; a) or P. falciparum (*Pf*‐IRBCs; c). (b,d) Mean values for volume, surface area and surface area to volume ratio for URBCs and *Pk*‐IRBCs (b) and *Pf*‐IRBCs (d). Ektacytometry profiles (0–20 Pa shear stress) for uninfected RBCs (squares) and RBCs infected with P. knowlesi (e) and P falciparum (f) trophozoites (97% 22‐ to 26‐hr and 99% 30–40‐hr postinvasion, respectively, circles). (g) The average EI ± SEM at 3 Pa (*n* = 3, minimum parasitemia 88%; 22–32 h and 30–40 h post invasion for P. knowlesi and P. falciparum, respectively). (h,i) RBCs infected with P. knowlesi and P. falciparum trophozoites were subjected (10 min) to increasing concentrations of sorbitol under iso‐osmotic conditions (h, *n* = 3) or buffers with different osmolarities (i, *n* = 4 and 3, respectively). Survival was measured by flow cytometry following Syto61‐labelling. Error bars indicate standard error of the mean (s.e.m.)

Our SBF‐SEM data suggest that the total volume of the infected RBC increases during intraerythrocytic development of P. knowlesi. Therefore, we sought a complementary method to measure the surface area and volume of live P. knowlesi‐infected RBCs taken directly from culture. We made use of a human erythrocyte microchannel analyser (HEMA) microfluidics device (Figure 8a,c), which can trap hundreds of RBCs and conform them into a defined regular geometry, permitting very accurate analysis of RBC surface area and volume (Gifford et al., 2003; Herricks, Antia, & Rathod, 2009; Lelliott et al., 2017).

Using the HEMA, we estimated that the average volume of an uninfected RBC is 88 ± 11 fl, and the average surface area is 147 ± 13 μm^2^ (Figure 8b; Figure S1), in good agreement with previous reports (Gifford et al., 2003; Hanssen et al., 2012; Park et al., 2008). By contrast, the average volume of late trophozoite P. knowlesi‐infected RBCs was 106 ± 16 fl (a 20% increase), whereas the surface area was 163 ± 23 μm^2^ (an 11% increase). The surface area: volume ratio (SA:V) decreased by ~8% in P. knowlesi‐infected RBCs compared with uninfected RBCs from the same culture. By contrast, we observed no change in the volume or surface area of RBCs infected with P. falciparum trophozoites (Figure 8d).

We used microfluidics‐based ektacytometry to measure cellular deformability at shear stresses from 0–20 Pa (Figure 8e). The elongation index (EI) of mature stage P. knowlesi‐infected RBCs (22 to 26‐hr postinfection; 97% parasitemia) was substantively decreased compared with uninfected RBCs, though less compromised than that of mature stage P. falciparum‐infected RBCs (30 to 40‐hr postinfection; 99% parasitemia; Figure 8f). These data indicate that the parasite‐induced changes to the RBC membrane substantively compromise its deformability. The combination of increased rigidity and decreased surface area: volume ratio would be expected to compromise passage of infected RBCs through the circulation (Diez‐Silva, Dao, Han, Lim, & Suresh, 2010; Safeukui et al., 2012).

### 2.8 P. knowlesi and P. falciparum‐infected RBCs exhibit differentially modified permeability properties

P. falciparum induces new permeability pathways (NPPs) in the host RBC membrane that are thought to play important roles in the delivery of nutrients and the efflux of waste (Lew, Tiffert, & Ginsburg, 2003; Nguitragool et al., 2011). Consequently, P. falciparum‐infected RBCs are susceptible to swelling and lysis upon exposure to 5% sorbitol (a substrate for the NPP). To further investigate the basis for the swelling of mature stage P. knowlesi‐infected RBCs, we examined the susceptibility to lysis upon exposure to solutes that can pass through the NPP. Although P. falciparum *‐*infected RBCs are rapidly lysed, P. knowlesi *‐*infected RBCs withstand resuspension in 5% sorbitol (Figure 8h), indicating that P. knowlesi lacks NPP or has NPPs with a different substrate specificity to those of P. falciparum.

We also examined the susceptibility of P. knowlesi‐infected RBCs to hypo‐osmotic lysis. P. falciparum and P. knowlesi‐infected RBCs were resuspended in buffers at different osmolarities. P. knowlesi‐infected RBCs exhibited a higher sensitivity to hypo‐osmotic lysis (Figure 8i), consistent with the observed swelling of mature stage P. knowlesi‐infected RBCs.

## 3 DISCUSSION

Here, we present the first detailed ultrastructural examination of P. knowlesi at different stages of development in mature human RBCs. We examined the cleft‐like structures, resembling P. falciparum Maurer's clefts and TV features that wrap around regions of host cell cytoplasm, similar to the P. falciparum TVN. They are assembled shortly after invasion, indicating that their function is required at all stages of intraerythrocytic development. Some of the TV structures are closely associated with the PVM and with invaginations of the RBC cytoplasm into the parasite cytoplasm, suggesting that they may play a role in generating the phagotrophic structures involved in the uptake of host cell cytoplasm. In the mature P. knowlesi trophozoite, structures with an electron‐lucent lumen bulge and bleb from the PVM. These structures are reminiscent of structures that have been observed in P. falciparum‐infected RBCs (Aikawa, Uni, Andrutis, & Howard, 1986; Atkinson & Aikawa, 1990) and may represent sites where secreted proteins accumulate prior to export (McMillan et al., 2013).

Nuclear division is initiated ~22 hr after invasion, producing up to 12 daughter merozoites. As division continues, the nuclei elongate, and apical organelles accumulate at the tip of the daughter cell, which faces towards the cell periphery. The mother cell adopts a crenated aspect as the plasma membrane develops around the individual merozoites, and the DV and other cellular contents contract to a central RB. Each of the daughter merozoites remains connected to the RB via a thin neck of cytoplasm until the final stages of development. In very mature schizonts, the PVM is breached. This is consistent with studies in P. falciparum suggesting that the PVM is permeabilised prior to rupture of the infected RBC (Hale et al., 2017).

Uptake of host cell haemoglobin occurs soon after invasion and involves both classical cytostome‐mediated endocytosis and an alternative phagocytic mechanism. The cytostomes exhibit an inner diameter of ~90 nm and outer diameter of ~120 nm, equivalent to that of P. falciparum cytostomes (Abu Bakar, Klonis, Hanssen, Chan, & Tilley, 2010; Lazarus, Schneider, & Taraschi, 2008). In addition, nonclassical pores of ~400 nm open into larger invaginations. Although P. falciparum mainly employs cytostome‐dependent uptake of host cell cytoplasm, large cytostome‐independent invaginations, called phagotrophs, are occasionally observed in trophozoite stage parasites (Abu Bakar et al., 2010; Elliott et al., 2008; Hanssen et al., 2011; Lazarus et al., 2008). P. knowlesi appears to utilise phagotroph‐like structures as an important component of the haemoglobin uptake process. The persistence of very large invaginations from the early ring stage to the early schizont stage suggests that the process for budding of these structures is relatively inefficient in P. knowlesi, potentially underpinning the volume control problems (see below).

The remarkable deformability and durability of the plasma membrane of RBCs is essential for survival in circulation and the ability to navigate small capillaries and splenic sinuses (Mohandas & Gallagher, 2008). We found that the ability of P. knowlesi trophozoite‐infected RBCs to elongate in response to a shear force was markedly compromised compared with uninfected RBCs. This is consistent with a recent study using ektacytometry and micropipette aspiration, which revealed increased rigidity of infected and uninfected RBCs in blood samples collected from adult patients with severe knowlesi malaria, reaching levels similar to those observed for patients with severe falciparum malaria (Barber et al., 2018).

Knob deposition provides a major contribution to increased membrane rigidity in P. falciparum‐infected RBCs (Glenister, Coppel, Cowman, Mohandas, & Cooke, 2002; Zhang et al., 2015). P. knowlesi‐infected RBCs lack knobs, and it is interesting to consider the molecular basis for the decreased deformability. SEM imaging of the cytoplasmic surface of infected RBC membranes revealed proteinaceous deposits. Similarly, SEM of whole cells revealed raised “pimples” that showed a similar distribution to the deposits at the cytoplasmic surface. These protein deposits may strengthen and stiffen the horizontal linkages within the RBC membrane skeleton, which may in turn constrain the flexibility of the skeleton (Dearnley et al., 2016).

The host RBC surface area increases by ~11% upon P. knowlesi‐infection, and it is interesting to consider the origin of the extra material. SEM of whole trophozoite P. knowlesi‐infected RBCs reveals the presence of caveolar pits, whereas electron tomography revealed small vesicles apparently budding from the Sinton‐Mulligan's clefts. Fusion of vesicles with the RBC membrane may deposit sufficient phospholipids to drive the surface area expansion. We observed only a modest (4.5%) increase in membrane skeleton network branch length in P. knowlesi‐infected RBC relative to uninfected RBCs. The degree of stretching of the membrane skeleton is less dramatic than that reported for P. falciparum trophozoite‐infected RBC, which has been estimated to be from 8% (Nunez‐Iglesias et al., 2018) to ~50% (Shi et al., 2013). The stretching of the membrane skeleton is insufficient to account for the increased surface area. It is possible that reorganisation of higher‐order spectrin oligomers (Nans et al., 2011) could provide material for the observed surface area increase.

Intraerythrocytic malaria parasites face the challenge of expanding their own volume while limiting the swelling of the host cell. In P. falciparum, volume control is complicated by the induction of NPPs that play roles in the delivery of nutrients and in the efflux of waste products (Lew et al., 2003; Martin & Kirk, 2007; Nguitragool et al., 2011; Saliba et al., 2006). P. falciparum tackles the problem of volume control by tightly coupling parasite growth and haemoglobin digestion (Hanssen et al., 2012) and by effluxing amino acids (Lew et al., 2003). P. falciparum schizonts occupy ~43% of the uninfected RBC volume (Hanssen et al., 2012), but we found that the P. falciparum‐infected RBC volume changes little. This finding agrees with several previous studies of infected RBC volume (Esposito et al., 2010; Hanssen et al., 2012; Herricks et al., 2011), although another study (Waldecker et al., 2017) found that P. falciparum *‐*infected RBCs become more spherical. By contrast, mature P. knowlesi‐infected RBCs swell to ~120% of the volume of uninfected RBCs. P. knowlesi‐infected RBCs are not susceptible to sorbitol lysis, suggesting that they lack NPP—although, further work is needed to confirm this finding. Instead, we suggest that the swelling may be due to inefficient degradation of host cell haemoglobin, as indicated by the persistence of the large invaginations. The baseline swelling of P. knowlesi‐infected RBCs may explain their increased sensitivity to hypo‐osmotic lysis. Taken together, our data suggest that P. knowlesi lacks efficient volume control mechanisms, which might be exploited by the use of drugs that target osmotic regulators (Dennis, Lehane, Ridgway, Holleran, & Kirk, 2018; Rottmann et al., 2010).

Decreased surface area to volume ratio is a major parameter leading to splenic entrapment (Safeukui et al., 2012). Accordingly, swelling of P. knowlesi‐infected RBC could be responsible for sequestration of mature stage P. knowlesi‐infected RBCs in small capillaries. Autopsy of a fatal knowlesi malaria case revealed brain capillary congestion (Menezes et al., 2012). Similarly, impaired microcirculatory flow has been observed in P. knowlesi infections of rhesus macaques—unnatural hosts who develop severe and fatal disease (Knisely et al., 1964). A recent study suggested that impaired deformability of both P. knowlesi‐infected RBCs and bystander uninfected RBCs may contribute to microvascular accumulation, impaired organ perfusion, and anaemia (Barber et al., 2018). Our data indicate that swelling of infected RBCs may also contribute to impaired rheology.

In summary, we have provided a detailed atlas of the ultrastructure of P. knowlesi developing in human RBCs, showing that during its ~32 hr lifecycle, it degrades host cell haemoglobin using unusual phagotrophic structures and divides to form ~12 daughter cells. It elaborates a variety of membranous structures in the host RBC and modifies the ultrastructure and the physical properties of the host RBC membrane, resulting in host cell swelling to a point that compromises the rheological properties of infected RBCs. The work raises questions with respect to the mechanism for obtaining nutrients in the apparent absence of NPPs and points to a need to further investigate the ultrastructure and rheological properties of P. knowlesi in natural infections of humans.

## 4 EXPERIMENTAL PROCEDURES

### 4.1 Culture of P. knowlesi

P. knowlesi A1‐H.1 strain (Moon et al., 2013) was cultured in O+ human RBCs (5% haematocrit) in complete culture media containing pooled human serum (5%), AlbuMAX™ II (5%), 200 μM hypoxanthine, 10 mM D‐glucose (Sigma) and 20 μg/ml gentamicin (Sigma). Parasitemia was routinely maintained below 5%.

Mature stage P. knowlesi‐infected RBCs were enriched from a culture by magnetic separation (Fivelman et al., 2007). The purified mature stages were resuspended at ~60% parasitemia with fresh RBCs and incubated for 7 hr with shaking. Mature stages were further depleted by a second passage through the magnet and an aliquot was taken as the 0 to 7‐hr window (~15% parasitemia). Additional aliquots were maintained in culture for a further 7 hr, then magnet purified to generate the 14 to 21‐hr window (~60% parasitemia) or returned to culture for a further 7 or 14 hr to generate the 21 to 28‐hr and 28‐ to 35‐hr windows.

### 4.2 Cell sorbitol sensitivity and osmotic fragility analysis

To assess comparative sorbitol sensitivity, P. knowlesi *‐* and P. falciparum‐infected RBCs were incubated with iso‐osmotic buffers of increasing sorbitol concentrations for 10 min at 37°C. For osmotic fragility analysis, cells were pelleted and resuspended in buffers of decreasing osmolarity, where 300 mOsm is physiological. Cells were centrifuged at 800 *g* for 90 s, washed, and labelled with Syto‐61 (Fu, Tilley, Kenny, & Klonis, 2010). Flow cytometry readouts were corrected for background signal from uninfected RBCs and normalised to the respective infected RBC control, which was taken as 100% survival.

### 4.3 Preparation of sheared membranes

Coverslips were sequentially treated with (3‐Aminopropyl)triethoxysilane, bis(sulfosuccimidyl) suberate crosslinker, and finally incubated with the ligand erythroagglutinating phytohemagglutinin (Shi et al., 2013). Parasites were enriched from culture at the required age of development and immobilised on the functionalised glass slides via a 2–3 min incubation at room temperature (RT). Reference images of cells stained for RNA using 1 μM Syto 61 in phosphate buffered saline (PBS) for 20 min were collected before shearing to facilitate identification of P. knowlesi *‐*infected RBCs. Bound RBCs were allowed to stand for 30 min prior to shearing with ~30 ml of 5P8–10 hypotonic buffer (5‐mM Na_2_HPO_4_/NaH_2_PO_4_, 10‐mM NaCl, pH 8) applied at a glancing angle across the bound cells using a 30 ml syringe with a 21‐G needle. The membrane discs were immediately fixed with 2.5% glutaraldehyde for 1.5 hr before dehydration in a series of ethanol: water mixtures and drying in air from 100% ethanol.

To identify Cv in the SEM images, sheared infected RBCs were fixed with 4% paraformaldehyde and 0.0065% glutaraldehyde for 20 min, rinsed with PBS, and incubated in 3% bovine serum albumin (BSA) in PBS with rabbit anti *Pv*PHIST‐81 (10 mg/ml, 1:2000) for 1 hr. Membranes were washed in PBS before incubation with a goat anti‐rabbit AlexaFluor647 secondary antibody (1:500) in 3% BSA for 1 hr. To facilitate correlative imaging, the RBC membranes were labelled with mouse anti‐α‐spectrin (Abcam) and goat anti‐mouse AlexaFluor488 (1:500, 1 hr each) in 3% BSA. A final PBS wash was applied before imaging at 100× magnification (widefield fluorescence, DeltaVision Elite). After imaging, this sample was fixed with glutaraldehyde and dehydrated before critical point drying (Leica EM CPD300).

Dried samples were gold coated on the rotating mount of a Dynavac SC100 sputter coating instrument for 35 s using a 25 mA current, measuring ~0.2 nm thickness on the quartz crystal microbalance. The coating procedure was optimised to prevent undercoating or overcoating, which compromises skeleton tracing.

SEM images were recorded using the Everhart‐Thornley Detector (ETD) detector of an FEI Teneo instrument (in “Optiplan” mode) with a working distance of 5 mm, a beam current of 50 pA and a 2 kV accelerating voltage. For skeleton analysis, multiple images in each of multiple cells were recorded at 200,000–250,000 magnification.

### 4.4 Cell deformability analyses

Mature stage P. knowlesi (22–32 hr) or P. falciparum (30–40 hr) were magnet purified and washed in PBS (final parasitemia of 88–99%). Pelleted samples (10 μl) were thoroughly mixed with 500 μl of 6% 360 kDa polyvinylindone in PBS. The EI was measured in a RheoScan Ektacytometer (Shin, Ku, Park, & Suh, 2005). Measurements were acquired over the 0–20 Pa range.

### 4.5 Microfluidics‐based analysis of cellular dimensions

HEMA (Gifford et al., 2003) microfluidic devices were fabricated using established procedures (Lake et al., 2015) at the Melbourne Centre for Nanofabrication and bound to clean coverslips after plasma treatment to ensure tight bonding (Lelliott et al., 2017). P. knowlesi‐infected RBCs were introduced into the channels at a flow pressure of 180–250 Pa, and trapped cells were imaged under flow (Gifford et al., 2003). Measurements of cell position within the channels were performed using a custom plugin written in ImageJ (NIH Image/ImageJ). Code written in R was applied to calculate volume, surface area, and surface area to volume ratio (SA:V) from measurements of cell position using the known geometry of the channels. (Plugin and R code are available upon request).

### 4.6 Serial block‐face scanning electron microscopy

The reduced osmium‐thiocarbohydrazide‐osmium staining method was described previously (Hanssen et al., 2013; Parkyn Schneider et al., 2017). In short, parasite pellets were fixed with 2.5% glutaraldehyde in PBS for 1 hr at 4°C. Agarose‐embedded cells were stained in ferrocyanide‐reduced osmium tetroxide in 0.15 M cacodylate buffer for 1 hr on ice. After washing, the cells were incubated with freshly prepared 1% thiocarbonhydrazide solution in H_2_O for 20 min at RT. The cells were then further stained with 2% osmium tetroxide in H_2_O for 30 min at RT. Subsequently, the cells were *en‐bloc* stained with 1% uranyl acetate overnight followed by Walton's lead aspartate for 30 min at 60°C. The cells were dehydrated in a graded series of ethanol‐H_2_O, followed by progressive infiltration with EPON resin.

After polymerisation, a 200 × 200 × 200 μm resin block was trimmed using an ultramicrotome (Leica EM UC7, Leica Microsystems). The block was mounted on a microtome stub using silver glue and further cleaned by diamond knife after gold coating. The serial images (every 50 nm) were collected using a SBF‐SEM, equipped with an in‐chamber diamond knife (Teneo VolumeScope, FEI Company), using back scattered electron signals at 3 kV, under low vacuum conditions. The pixel size of each image in the stack was 5 nm.

### 4.7 SBF‐SEM image analysis and volume rendering

Serial sections were contrasted and aligned using IMOD software (Boulder Laboratory for 3D Electron Microscopy of Cells). The regions of interest were segmented and reconstructed into 3D models, using semi‐automatic methods described previously (Maiorca et al., 2012). Briefly, the pixel size of the raw images was binned to 20 nm in the xy direction, then, images were subjected to bandpass filter and Gaussian smoothing before a threshold value was manually selected from each reconstructed tomogram (Parkyn Schneider et al., 2017). Cellular components were rendered as individual objects, which were separated by rough bounding areas drawn manually on 2D sections. The components were labelled with different colours.

Manual segmentation was used for rendering some features where a more accurate volume and surface area estimate were required. The IMOD slicer tool was used to orient image stacks at the maximum length or width of the object of interest, and the relative distances from the ends were measured using open contours in IMOD. The correct positioning of the open contours was confirmed on 3D models.

### 4.8 Transmission electron microscopy and electron tomography

Permeabilization with EqtII was performed as described previously (Jackson et al., 2007). Briefly, cells were prefixed with 2% paraformaldehyde in PBS for 10 min at RT, followed by incubation of 47 ng/μl EqtII for 6 min. The cells were pelleted at 1,400 *g* for 2 min. For conventional staining, the cells were fixed with 2.5% glutaraldehyde in PBS for 1 hr at 4°C. Cells were preembedded in agarose and fixed in 2% osmium tetroxide in 0.15 M cacodylate buffer for 1 hr at RT. Subsequently, the cells were dehydrated in a graded series of ethanol‐H_2_O mixtures, followed by progressive infiltration with EPON resin and embedding; 70 nm sections were prepared using an ultramicrotome (Leica EM UC7, Leica Microsystems). For electron tomography of permeabilised parasites, 450 nm sections were collected on single slot grids. For electron tomography of intact cells, 300 nm serial sections were collected. The conventionally stained sections were poststained with 7% uranyl acetate in methanol and Reynold's lead citrate. Thin sections were observed on a transmission electron microscope at 200 kV (Tecnai G2 F30, FEI), and tilt series of thick sections were collected at 300 kV. Tilt series were aligned in IMOD. The regions of interest were segmented on the tomograms and presented in 3D models.

### 4.9 Quantification and statistical analysis

Statistical analyses were performed using the GraphPad Prism 7 software package. *p* values were calculated using unpaired *t* tests, with details provided in the figure legends. For quantification of the membrane skeleton branch length, the program Skan was applied to unmodified SEM images with a crop range of 20 pixels, offset of 0.075, Gaussian smoothing radius 0.1, and a threshold radius of 5 × 10^−8^. Three separate experiments were performed, analysing a total of 29 uninfected RBCs and 45 infected RBCs. To overlay fluorescence and SEM data, the “landmark correspondences” plugin in FIJI was used to align features between the data sets (minimum of 10 points per image pair) before merging the channels.

## Supporting information


**Figure S1.** **Quantification of** ***P. knowlesi*** **morphological parameters.** (a) Parasite diameter increases steadily during development. (b,c) The number and size of clefts remains roughly constant during development. (d) The number of TV increases during development. (e) Nuclear division is initiated ~21 h post‐invasion leading to an increase in total nuclear volume. (f) The parasite surface area to volume ratio decreases with parasite age. Data represent mean values and standard errors. Unpaired t‐test; ** *P* < 0.01.

**Figure S2.** **Caveolae in** ***P. knowlesi*** **‐infected RBCs labelled with anti‐*Pv*PHIST‐81.** Late trophozoite stage P. knowlesi‐infected RBCs were immobilized onto glass slides, sheared and fixed. Caveolae were labelled with *Pv*PHIST‐81 antiserum followed by AlexaFluor 647‐labeled secondary antibody. Corresponding SEM (a, left panel) and widefield fluorescence (a, centre panel) images were recorded of the same regions and were overlaid using landmark correspondences (a, right panel). (b) Bright puncta remain in the SEM image for regions showing high intensity in the (hydrated) florescence image. The absence of defects in the membrane surrounding these puncta (c, d) suggests that the caveolae collapse upon adhesion to the coverslip surface, with remaining invaginated material contracting into a raised aggregate upon dehydration for SEM imaging. Scale bars: 500 nm.

**Video S1.** Rotating models constructed from SBF‐SEM of P. knowlesi A1‐H.1 strain‐infected RBCs at 0–7 h post‐invasion, showing the initial formation of invaginations and exomembrane features. Two cells are featured. The following features are rendered: PVM, pale yellow; nuclei, pink; invagination, yellow; RBC, translucent red; clefts, white; TV, cyan. Scale bar: 1 μm.

**Video S2.** 2D translations through virtual sections of the SBF‐SEM data used to generate the models in Video S1. Scale bar: 1 μm.

**Video S3.** Rotating models constructed from SBF‐SEM of P. knowlesi‐infected RBCs at 14–21 h post‐invasion, showing mature DVs. Two cells are featured. The following feature are rendered: PVM, pale yellow; nuclei, pink; invagination, yellow; DV, green; RBC, translucent red; clefts, white; TV, cyan. Scale bar: 1 μm.

**Video S4.** 2D translations through virtual sections of the SBF‐SEM data used to generate the models in Video S3. Scale bar: 1 μm.

**Video S5.** Rotating models constructed from SBF‐SEM of P. knowlesi‐early schizont infected RBCs (28–35 h post‐invasion), showing nuclear division, and formation of daughter merozoites. Two cells are featured. The following feature are rendered: PVM, pale yellow; blebs, red; nuclei, pink; remnant body, green; lipid bodies, blue; RBC, translucent red; clefts, white; TV, cyan. Scale bar: 1 μm.

**Video S6.** 2D translations through virtual sections of the SBF‐SEM data used to generate the models in Video S5. Scale bar: 1 μm.

**Video S7.** Rotating models constructed from SBF‐SEM of P. knowlesi‐infected RBCs at 21–28 h post‐invasion, showing PVM bulges and initial stages of nuclear division. Two cells are featured. The following feature are rendered: PVM, pale yellow; PVM bulges, fawn; nuclei, pink; invagination, yellow; DV, green; RBC, translucent red; clefts, white; TV, cyan. Scale bar: 1 μm.

**Video S8.** 2D translations through virtual sections of the SBF‐SEM data used to generate the models in Video S7. Scale bar: 1 μm.

**Video S9.** Rotating models constructed from SBF‐SEM of P. knowlesi‐early schizont infected RBCs (28–35 h post‐invasion), showing nuclear division, and formation of daughter merozoites. Two cells are featured. The following feature are rendered: PVM, pale yellow; blebs, red; nuclei, pink; DV, green; invagination, yellow; lipid bodies, deep blue; RBC, translucent red; clefts, white; TV, cyan. Scale bar: 1 μm.

**Video S10.** 2D translations through virtual sections of the SBF‐SEM data used to generate the models in Video S9. Scale bar: 1 μm.

**Video S11.** Rotating models constructed from SBF‐SEM of P. knowlesi‐segmented schizont infected RBCs (28–35 h post‐invasion), showing cytokinesis, remnant body separation and PVM rupture. Two cells are featured. The following feature are rendered: PVM, pale yellow; blebs, red; nuclei, pink; remnant body, green; RBC, translucent red; clefts, white; TV, cyan. Scale bar: 1 μm.

**Video S12.** 2D translations through virtual sections of the SBF‐SEM data used to generate the models in Video S11. Scale bar: 1 μm.

**Video S13.** Rotating models constructed from electron tomograms of P. knowlesi‐segmented merozoite and free merozoite (28–35 h post‐invasion), showing organelles of young and mature merozoites. The following feature are rendered: PVM, pale yellow; rhoptries, blue; nuclei, pink; mitochondria, red; apicoplast, orange; ER, white; DG, blue‐green; remnant body, green. Scale bar: 500 nm.

**Video S14.** 2D translations through virtual sections of the electron tomography data used to generate the models in Video S13. Scale bar: 500 nm.
