# Supplementary figures and images for: Multimodal analysis of Plasmodium knowlesi‐infected erythrocytes reveals large invaginations, swelling of the host cell, and rheological defects
Source: Cell Microbiol. 2019 Feb 11;21(5):e13005. doi: 10.1111/cmi.13005 (PMC6593759; doi:10.1111/cmi.13005)

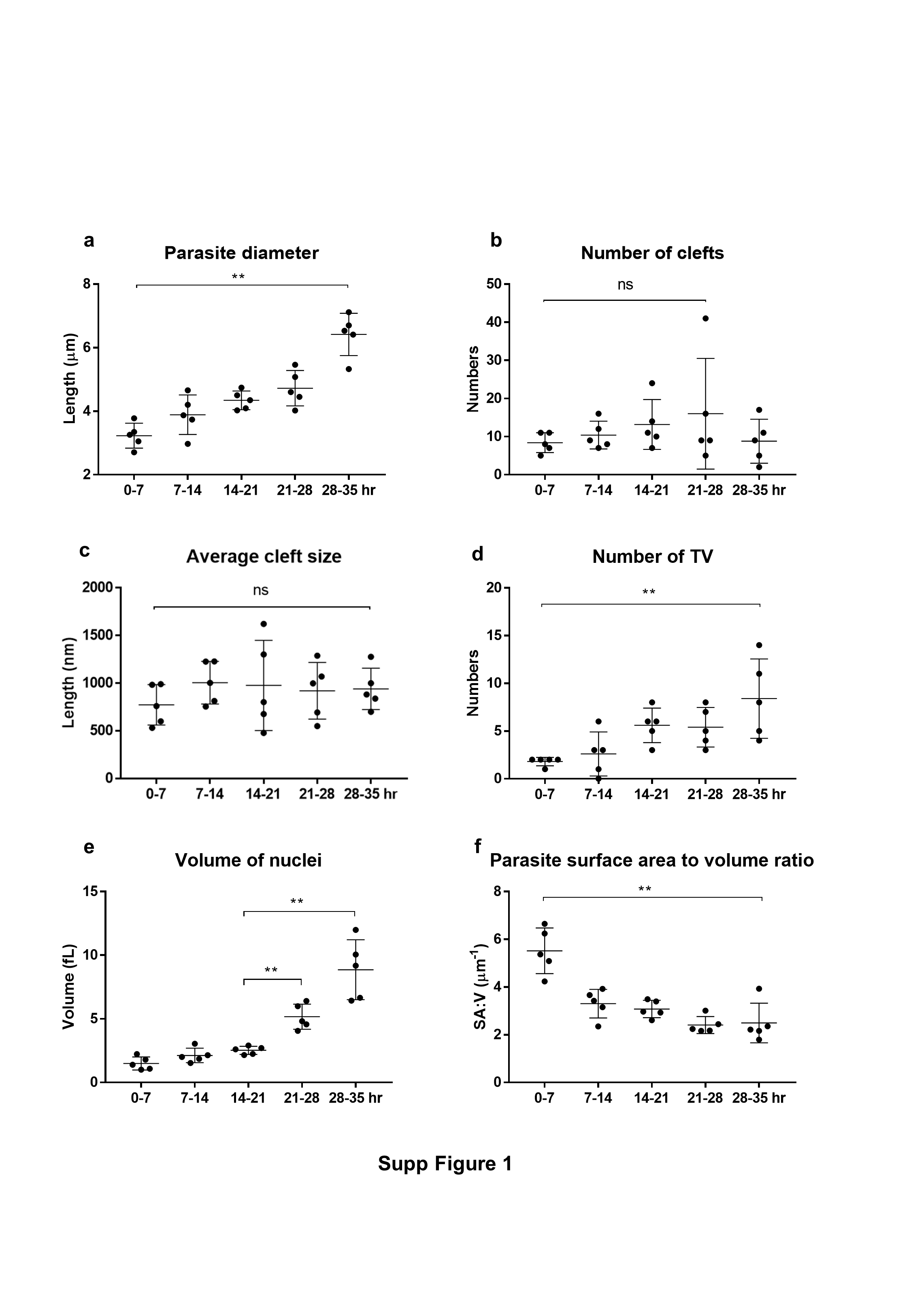

Supplement: Supplementary file 1 — Figure S1. Quantification of P. knowlesi morphological parameters. (a) Parasite diameter increases steadily during development. (b,c) The number and size of clefts remains roughly constant during development. (d) The number of TV increases during development. (e) Nuclear division is initiated ~21 h post‐invasion leading to an increase in total nuclear volume. (f) The parasite surface area to volume ratio decreases with parasite age. Data represent mean values and standard errors. Unpaired t‐test; ** P < 0.01. [file CMI-21-na-s001.TIF]

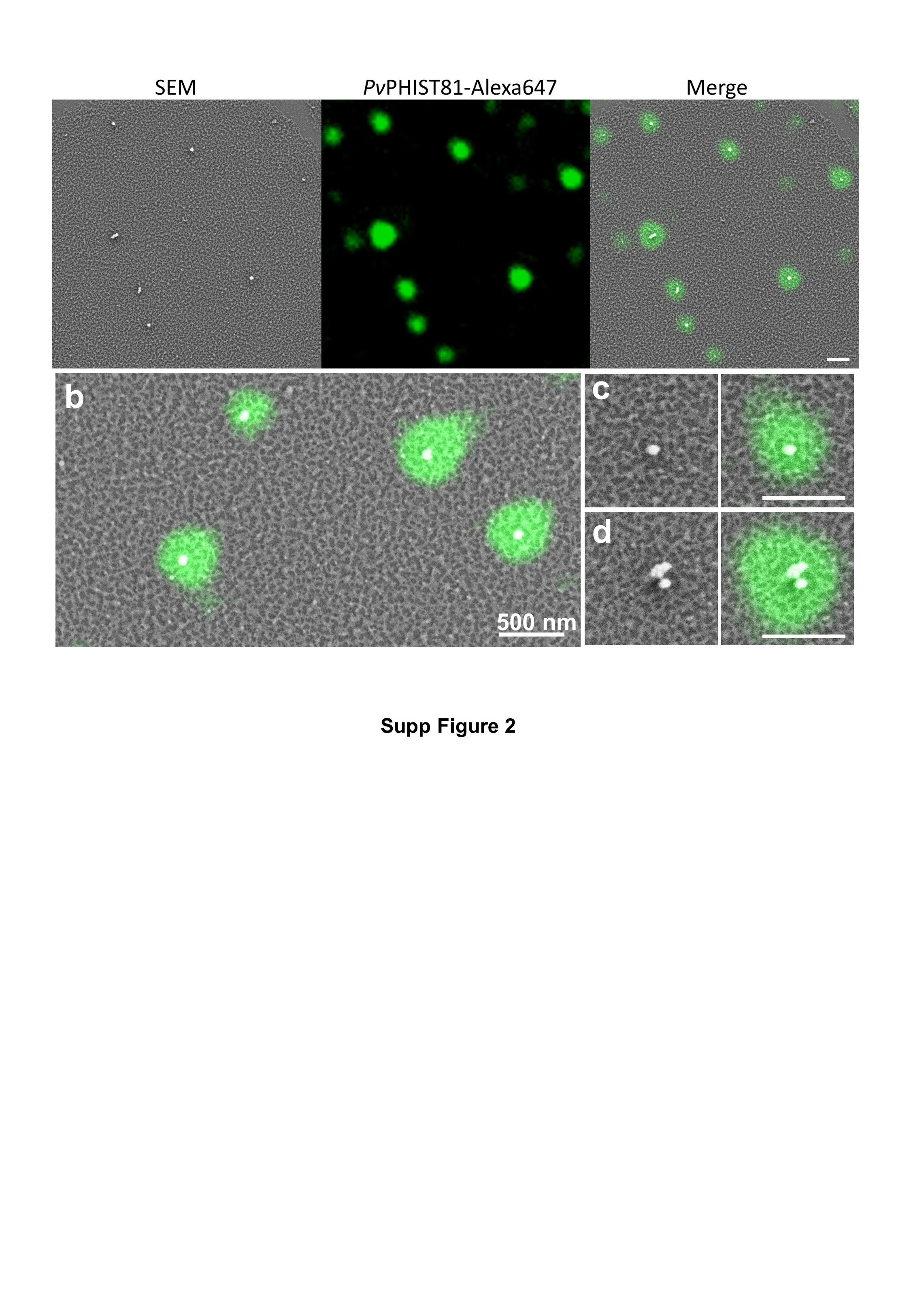

Supplement: Supplementary file 2 — Figure S2. Caveolae in P. knowlesi ‐infected RBCs labelled with anti‐PvPHIST‐81. Late trophozoite stage P. knowlesi‐infected RBCs were immobilized onto glass slides, sheared and fixed. Caveolae were labelled with PvPHIST‐81 antiserum followed by AlexaFluor 647‐labeled secondary antibody. Corresponding SEM (a, left panel) and widefield fluorescence (a, centre panel) images were recorded of the same regions and were overlaid using landmark correspondences (a, right panel). (b) Bright puncta remain in the SEM image for regions showing high intensity in the (hydrated) florescence image. The absence of defects in the membrane surrounding these puncta (c, d) suggests that the caveolae collapse upon adhesion to the coverslip surface, with remaining invaginated material contracting into a raised aggregate upon dehydration for SEM imaging. Scale bars: 500 nm. [file CMI-21-na-s002.TIF]
